# A quantitative model based on gross tumor volume of gastric adenocarcinoma corresponding to N-stage measured at multidetector computed tomography for preoperative determination of resectability: A case control study

**DOI:** 10.3389/fonc.2022.1001593

**Published:** 2022-10-05

**Authors:** Zi-yi Yu, Dan Gao, Zhao Tang, Hai-ying Zhou, Jing Ou, Ke-ying Li, Xiao-qian Chen, Dan Yang, Lin-li Yan, Rui Li, Xiao-ming Zhang, Tian-wu Chen

**Affiliations:** Medical Imaging Key Laboratory of Sichuan Province, and Department of Radiology, Affiliated Hospital of North Sichuan Medical College, Nanchong, China

**Keywords:** stomach neoplasms, adenocarcinoma, tomography, X-ray computed, volume, ROC curve

## Abstract

**Purpose:**

To develop and validate a quantitative model based on gross tumor volume (GTV) of gastric adenocarcinoma (GA) corresponding to N-stage measured at multidetector computed tomography (CT) for preoperative determination of resectability

**Materials and methods:**

493 consecutive patients with confirmed GA undergoing contrast-enhanced CT two weeks before treatments were randomly enrolled into the training cohort (TC, n = 271), internal validation cohort (IVC, n = 107) and external validation cohort (EVC, n = 115). GTV was measured on CT by multiplying sums of all tumor areas by section thickness. In TC, univariate and multivariate analyses were performed to select factors associated with resectability. Receiver operating characteristic (ROC) analysis was to determine if N-stage based GTV could identify resectability. In IVC and EVC, unweighted Cohen’s Kappa tests were to evaluate performances of the ROC models.

**Results:**

According to univariate analysis, age, cT stage, cN stage and GTV were related to resectability in TC (all *P*-values < 0.05), and multivariate analysis suggested that cN stage and GTV were independent risk factors with odds ratios of 1.594 (95% confidence interval [CI]: 1.105–2.301) and 1.055 (95%CI: 1.035–1.076), respectively. ROC analysis in TC revealed the cutoffs of 21.81, 21.70 and 36.93 cm^3^ to differentiate between resectable and unresectable cancers in stages cN_0-3_, cN_2_ and cN_3_ with areas under the curves of more than 0.8, respectively, which was validated in IVC and EVC with average Cohen k-values of more than 0.72.

**Conclusions:**

GTV and cN stage can be independent risk factors of unresectable GA, and N-stage based GTV can help determine resectability.

## Introduction

The incidence of gastric cancer has gently declined in recent years, but its mortality remains high and survival rate is still not optimistic worldwide (1, 2). Gastric adenocarcinoma (GA) is the most common pathological type (3). In clinical practice, radical surgical resection with lymphadenectomy constitutes the major and necessary treatment strategy at present (4, 5). Although the preoperative therapies, including chemotherapy, radiotherapy, targeted therapy and immunotherapy, are widely used in the treatment, these strategies are still aiming at increasing the opportunity for curative resection ultimately, which brings more chances for patients to win a better overall survival (6, 7). It’s crucial to determine resectablity of GA to achieve the best prognosis of patients after precise treatments.

Computed tomography (CT) can reflect the manifestation of thickened gastric wall. This technique is widely used in the diagnosis, staging, therapy response evaluation and follow-up of patients with gastric cancer (8–10). With the rapid development of imaging technology, gross tumor volume (GTV) of GA obtained on CT or magnetic resonance imaging has become a hot topic in radiology researches in recent years. There are some studies about the relationship between GTV and N stage, lymphovascular invasion, or radiotherapy response in GA based on imaging technology to date (11–13). Jiang et al. reported that GTV of resectable adenocarcinoma of gastroesophageal junction could increase with the number of metastatic lymph nodes (11). Chen et al. suggested that tumor volume of resectable gastric cancer could predict lymphovascular invasion (12). Dębiec et al. suggested that the GTV delineation might be affected by 18-fluorodeoxy-glucose positron emission tomography-computed tomography in gastric cancer patients undergoing radiotherapy (13). According to the previous studies (11, 14), the larger GTV of GA, the more advanced N stage. We can presume that if GTV measured at CT might help determine the resectability. To our knowledge, there were no reports focusing on the association of GTV with the resectability of GA. Therefore, the aim of our study was to develop and validate a quantitative model based on GTV of GA corresponding to N-stage measured at multidetector CT for preoperative determination of the resectability.

## Materials and methods

### Patients

This retrospective study was approved by the institutional ethics committee of our hospital. All patients signed informed consent before participating in this study.

According to the National Comprehensive Cancer Network (NCCN) guidelines of gastric cancer (15), the definition of unresectable gastric cancer consisted of the following two requirements: (a) gastric cancer with locoregionally advanced cases (e.g., disease infiltration of the root of the mesentery, or para-aortic lymph node, or major vascular structures other than splenic vessels); or (b) gastric cancer with distant metastasis or peritoneal seeding. If the tumor did not meet the above definitions of unresectable GA, this tumor could be considered resectable.

The inclusion criteria were as follows: (a) no tumor-related treatment (e.g., chemotherapy or radiotherapy) was performed before patients underwent abdominal contrast-enhanced CT scans; (b) GA was regarded unresectable and resectable according to the previous NCCN guidelines based on CT findings (16); and (c) the surgical cut edges in all resected specimens were not infiltrated by the tumor. From December 2018 and January 2021, a total of 519 consecutive patients with GA diagnosed by endoscopic biopsy from two centers (Affiliated Hospital of North Sichuan Medical College, and Nanchong Center Hospital) were enrolled into this study. A total of 26 patients were excluded from this study because of the following reasons: (a) patients had other malignant tumor history (n = 6); (b) patients with incomplete data on CT descriptors or/and clinical records (n = 4); or (c) patients with primary tumor which could not be identified on CT (n = 16). Consequently, 493 patients including 356 with resectable disease and 137 with unresectable disease were involved in our study. Of the 356 patients with resectable tumors, 345 patients with primary resectable tumors did not receive neoadjuvant therapy but surgery; and the remained 11 patients received neoadjuvant therapy after CT and before surgical treatment, the tumors shrank on therapy, the cases changed to resectable tumors, and these patients subsequently underwent successful surgery. All the enrolled patients were randomly divided into a training cohort (TC, n = 271), an internal validation cohort (IVC, n = 107) and an external validation cohort (EVC, n = 115). In addition, the patients in the previous TC and IVC were collected from December 2018 to January 2021 at Affiliated Hospital of North Sichuan Medical College, and the former 271 were randomly enrolled into the TC and the remained 107 were enrolled into the IVC. The patients in the EVC were recruited from March 2020 to January 2021 at Nanchong Center Hospital. All participants underwent whole abdominal contrast-enhanced CT scanning 2 weeks before the surgical treatment. All tumors in patients who received the surgical treatment were confirmed resectable according to the surgical observations. The clinical, surgical and pathological data were collected from the hospital information system. On the basis of American Joint Committee on Cancer (AJCC) TNM staging classification for carcinoma of the stomach (17), the tumor anatomical distribution, T stage, N stage of GA are listed in **Table 1**.

**Table 1:** Clinical information in the training cohort, and internal and external validation cohorts.

Variable	TC (n = 271)	IVC (n =107)	EVC (n =115)
Number of patients (resectable vs. unresectable)	271 (200 vs. 71)	107 (80 vs. 27)	115 (76 vs. 39)
Sex: male vs. female	175 vs. 96	73 vs. 34	81 vs. 34
Age, median (range) in year	62 (22-86)	62 (31-80)	63 (39-78)
Anatomical distribution			
Gastric antrum	148	71	64
Gastric body	94	31	42
Gastric antrum and body	29	5	9
cT stage			
cT_1_	37	11	7
cT_2_	53	18	21
cT_3_	109	57	59
cT_4_	72	21	28
cN stage			
cN_0_	81	25	13
cN_1_	42	17	6
cN_2_	61	29	52
cN_3_	87	36	44
GTV, mean ± SD (cm^3^)	34.78 ± 3.24	23.20 ± 2.64	28.47 ± 2.99

TC, training cohort; IVC, internal validation cohort; EVC, external validation cohort; GTV, gross tumor volume; and SD, standard deviation.

### Contrast-enhanced CT scans

All patients underwent contrast-enhanced imaging with 64-section multidetector computed tomography (MDCT) (LightSpeed VCT, GE Medical systems, USA). Before the CT scanning, at least 800 to 1000 ml of water was given orally to dilate the stomach. Patients were examined in the supine position. After a routine unenhanced scan, the contrast-enhanced CT acquisitions were started 25 s and 65 s after the injection of contrast medium (Omnipaque, Iohexol, GE Healthcare, USA) *via* a 20-G needle into an antecubital vein at a rate of 3.0 ml/s for a total of 70-100 ml tailored to body weight at the ratio of 1.5 ml/kg weight, followed by a 20 ml saline flush with a pump injector (Vistron CT Injection System, Medrad, USA). The arterial phase and portal venous phase images were acquired successively through the contrast-enhanced scanning. Scanning parameters for unenhanced and enhanced scans were 120 kV of peak voltage, 200 mA of tube current, rotation time of 0.5 s, collimation of 64 × 0.6 mm, pitch of 0.9, slice thickness of 2 mm, and matrix of 512 × 512 mm. Each examination was performed during one breath-hold at full suspended inspiration for 10-15 s. The range of CT scans was from the level of liver dome to that of pubic symphysis. The CT data were directly transferred to the General Electric Advantage Workstation 4.4 at a window width of 380 HU and a window level of 50 HU.

### Gross tumor volume measurement

**Figure 1:**
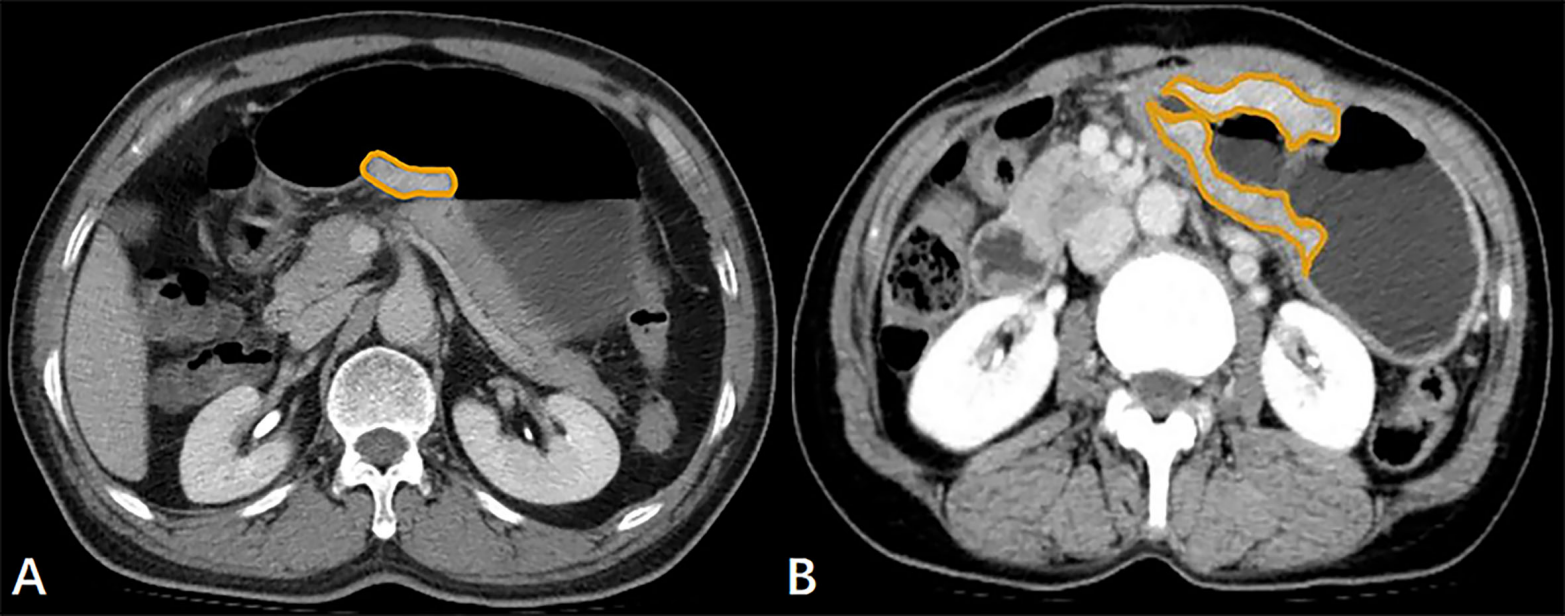
**(A)** In a 61-year-old male with resectable gastric adenocarcinoma, the initial preoperative abdominal contrast-enhanced computed tomography scans depict the gross tumor volume (GTV) obtained by manual sketching along the margin of the lesser curvature of the thickened stomach wall slice-by-slice, and the GTV is 9.94 cm^3^. **(B)** The transverse contrast-enhanced computed tomography scans in a 62-year-old female with unresectable gastric adenocarcinoma with lung metastasis show that the tumor area is manually drawn along the margin of the thickened gastric wall, and the GTV is 39.10 cm^3^.

The GTV of both resectable and unresectable GA lesions was measured on the above-mentioned workstation, obtained by multiplying the sum of all the tumor areas by the slice thickness according to the published report (18). When the stomach is dilated and the gastric wall is greater than 5 mm, it is considered to be abnormal gastric wall thickening caused by the tumor (19). Images obtained during portal venous phase of contrast-enhanced scans performed best in manifesting the thickening of stomach wall, so we selected the portal venous phase images to measure GTV. The contour of the GA focus was manually drawn along the visible margins of the tumor, avoiding air, water and stomach contents (**Figure 1**), and then the software automatically calculated the tumor area. The above analysis was repeated for each contiguous transverse sections until the whole tumor was covered on axial CT images. When it was difficult to accurately sketch along the contours of tumors based on the axial CT data, we obtained a coronal or sagittal reconstruction image for better delineation.

To verify the interobserver reproducibility of GTV measurement in TC, a radiologist with 2 years of practice in radiology (Observer 1) and a radiologist with 5 years of radiology expertise (Observer 2) worked independently to measure the GTV of all patients. To verify the intraobserver reproducibility of GTV measurement, Observer 1 carried out a repeated measurement one month later. Before the actual GTV measurements, a professor of radiology (the corresponding author, with 24 years of experience in abdominal radiology) trained them to identify and outline lesions accurately in 20 patients at random.

### Statistical analysis

All statistical analyses were performed using the SPSS statistic software (version 25.0 for Windows; SPSS, Chicago, IL, USA) and a *P*-value of less than 0.05 was considered statistically significant. The inter- and intraobserver reliability of GTV measurements was estimated *via* the intraclass correlation coefficient (ICC). ICC was assessed according to the following rating scheme (20): less than 0.5, poor reliability; between 0.5 and 0.75, moderate reliability; between 0.75 and 0.9, good reliability; and greater than 0.90, excellent reliability.

For TC, the univariate analysis was conducted to determine whether GTV and the clinical factors including gender, age, and cT and cN stages could be associated with the resectability of GA by Chi-square test or Fisher’s exact test. And then the significant factors identified on the univariate analysis were subjected to the multivariate analysis using the binary logistic regression analysis to clarify the independent risk factors of unresectability of GA. The Mann-Whitney U test was used to compare GTV corresponding to different cN stages between resectable and unresectable groups. If a significant difference was proved by the Mann-Whitney U test, the cutoff values of GTV were then determined with the receiver-operating characteristic (ROC) analysis for identifying the resectability. Finally, we adopted Unweighted Cohen’s Kappa test to evaluate the performance of the previous ROC models to determine resectability of GA in the IVC and EVC dataset. Cohen k value less than 0.20, 0.21 to 0.40, 0.41 to 0.60, 0.61 to 0.80 and greater than 0.81 were demonstrative of poor, moderate, good, very good and excellent agreements, respectively (21).

## Results

### Patterns of resectable and unresectable GA

In TC, there were 200 patients (73.8%, 200/271) with diagnosed resectable GA at the onset, while the remaining 71 patients (26.2%, 71/271) were unresectable cases. In the previous 71 patients with unresectable cancer, 9 (12.6%), 36 (50.7%), 10 (14.1%) and 16 (22.5%) cases had locoregional progress, distant metastasis, peritoneal seeding, and two or more previous patterns, respectively. As for the above two or more patterns in the 16 patients with unresectable cancer, there were 10 (62.5%) with locoregional progress and distant metastasis in liver, lung or bone; and 6 (37.5%) had peritoneal seeding in ovaries and distant metastasis in liver, lung or bone. In IVC and EVC, the percentages of resectable cases were 74.8% (80/107) and 66% (76/115), and the those of unresectable cases were 25.2% (27/107) and 34.0% (39/115), respectively. The distant metastasis consisted the main patterns of unresectable GA in IVC and EVC, and the corresponding percentages were 62.96% (17/27) and 61.5% (24/39), respectively.

### Intra- and interobserver reliability of GTV measurements in TC

The initial measurement of GTV (mean ± standard deviation) of the observer 1 was 34.78 ± 3.24 cm^3^ (range, 0.22–471.53 cm^3^) in TC. To assess the reproducibility of GTV measurement, the intra- and interobserver ICC values of GTV measurement were 0.986 (95% confidence interval [95%CI], 0.981–0.989) and 0.993 (95%CI, 0.991–0.994), respectively, each with a *P*-value less than 0.001, suggesting excellent repeatability of GTV measurements of observer 1 in TC. Ultimately, we used the initial GTV measurement by observer 1 for the further statistical analysis.

### Univariate analysis of GTV and possible clinicopathological factors associated with resectability of GA in TC

Both clinicopathological factors and GTV of GA related to the resectability in TC are listed in **Table 2**. According to univariate analysis, the resectability was associated with age, cT stage, cN stage and GTV. However, the results suggested that gender and anatomical distribution were unrelated factors in this study.

**Table 2:** Univariate analysis of clinicopathological factors and gross tumor volume correlated with resectability of gastric adenocarcinoma in the training cohort.

Parameter	Resectable group (n = 200)	Unresectable group (n = 71)	*P*-value
Mean Age (year)			0.016
≤61	90 (45.0)	35 (49.3)	
>61	110 (55.0)	36 (50.7)	
Sex			0.269
Male	133 (66.5)	42 (59.2)	
Female	67 (33.5)	29 (40.8)	
Anatomical distribution			0.14
Gastric antrum	120 (60.0)	28 (39.4)	
Gastric body	73 (36.5)	21 (29.6)	
Gastric antrum and body	7 (3.5)	22 (31.0)	
cT stage			<0.0001
cT_1_	37 (18.5)	0	
cT_2_	51 (25.5)	2 (2.8)	
cT_3_	79 (39.5)	30 (42.3)	
cT_4_	33 (16.5)	39 (54.9)	
cN stage			<0.0001
cN_0_	76 (38.0)	5 (7.0)	
cN_1_	37 (18.5)	5 (7.0)	
cN_2_	45 (22.5)	16 (22.5)	
cN_3_	42 (21.0)	45 (63.5)	
Gross tumor volume (cm³)			<0.0001
≤34.78	181 (90.5)	16 (22.5)	
>34.78	19 (9.5)	55 (77.5)	

Numbers in the bracket are percentages of patients.

### Multivariate analysis of GTV and possible clinicopathological factors associated with resectability of GA in TC

Based on the results of the univariate analysis, age, cT stage, cN stage and GTV of GA were selected as potential independent risk factors of unresectability, and the binary logistic regression analysis was conducted to determine the independent risk factors. Consequently, cN stage (*P* = 0.013; odds ratio [OR] = 1.594; 95%CI of 1.105-2.301) and GTV (*P* < 0.0001; OR = 1.055; 95%CI of 1.035-1.076) were the independent risk factors.

### Association of N-stage based GTV with resctability of GA in TC

Associations of N-stage based GTV with resectability of GA were analyzed by the Mann-Whitney U test. Considering the numbers of patients in stages cN_0_ and cN_1_ in the unresectable group were too small (n =10), we included patients with stages cN_2_ and cN_3_ in the individual Mann-Whitney U tests with the exception of patients in stages cN_0_ and cN_1_. The Mann-Whitney U tests showed that GTV in stages cN_0-3_, cN_2_ and cN_3_ could be higher in the unresectable group than in the resectable group in TC (all *P*-values < 0.0001).

### ROC analysis of N-stage based GTV to determine resectability of GA in TC

**Figure 2:**
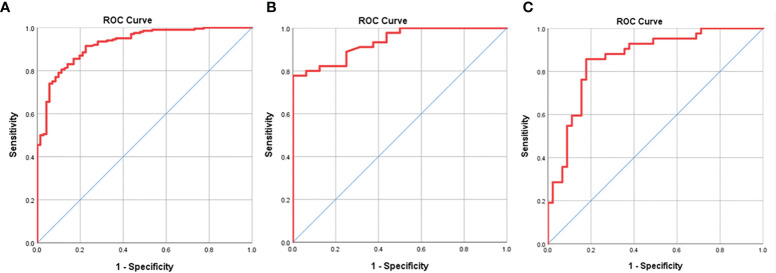
Receiver operating characteristic (ROC) analysis of N-stage based gross tumor volume (GTV) has been performed for estimating the resectability of gastric adenocarcinoma, and the ROC curves show that GTV can help judge the resectability of the tumor in stages cN_0-3_ **(A)**, cN_2_ **(B)** and cN_3_ **(C)** with the cutoff values of 21.83, 21.70 and 36.93 cm^3^, respectively.

The ROC analysis was executed to show the performance of N-stage based GTV of GA for the determination of resectability. At ROC analysis of all the 271 patients with cN_0-3_ disease in TC, we found that the N-stage based GTV cutoff of 21.81 cm^3^ could contribute to identify the resectability. And the threshold GTV values of 21.70 cm^3^ and 36.93 cm^3^ were enabled to identify the resectability in patients at stage cN_2_ and cN_3_, respectively. The areas under the ROC curve (AUC) were 0.925, 0.934 and 0.859 in estimating the resectability of GA in stage cN_0-3_, cN_2_ and cN_3_ (**Figure 2**), respectively. The AUC, sensitivity, specificity, positive predictive value, negative predictive value, and accuracy of GTV to identify the resectability are summarized in **Table 3**.

**Table 3:** Receiver operating characteristic analysis of N-stage based gross tumor volume for identifying resectability of gastric adenocarcinoma in the training cohort.

N categories	Cutoff (cm³)	AUC	Sen (%)	Spe (%)	PPV (%)	NPV (%)	ACC (%)
cN_0-3_	21.81	0.925	95.3	61.8	80.5	88.7	82.7
cN_2_	21.70	0.934	92.1	69.6	83.3	84.2	83.6
cN_3_	36.93	0.859	81.8	86.0	85.7	82.2	83.9

AUC, area under the receiver operating characteristic curve; Sen, sensitivity; Spe, specificity; PPV, positive predictive value; NPV, negative predictive value; and Acc, accuracy.

### Unweighted Cohen’s Kappa tests in IVC and EVC for validating performance of the ROC models

In order to validate the performance of the ROC models of N-stage based GTV for differentiating between resectable and unresectable GA lesions in stages cN_0-3_, cN_2_ and cN_3_, unweighted Cohen’s Kappa tests were performed in IVC and EVC according to the cutoff values obtained by the previous ROC analyses in TC. The tests revealed that the models obtained good agreements in validation cohorts as shown in **Table 4**.

**Table 4:** Unweighted Cohen’s kappa tests for validating the performance of the receiver operating characteristic models.

Categories	Cohen K value	
	IVC	EVC
cN_0-3_	0.858 (0.748-0.968)	0.815 (0.707-0.923)
cN_2_	0.813 (0.568-1.058)	0.847 (0.704-0.990)
cN_3_	0.776 (0.570-0.982)	0.720 (0.516-0.924)

IVC, internal validation cohort; EVC, external validation cohort; and values in the brackets are 95% confidence interval.

## Discussion

According to the NCCN guidelines of gastric cancer, the unresectable stomach tumor showed more aggressive behaviors than the resectable tumors did. Initially, our research revealed that the resectability of GA could be concerned with age, cT stage, cN stage and GTV according to the univariate analysis. Our multivariate analysis suggested that the cN stage and GTV could be independent risk factors of unresectability. Considering the previous two independent risk factors, we subsequently illustrated feasibility of N-stage based GTV to identify the resectability of GA.

As shown in our study, the cN stage and GTV could be independent risk factors of unresectability of GA. Some aggressive behaviors of gastric cancer, such as vascular infiltration, lymphatic permeation and depth of invasion have been proved to have relations with lymph node metastasis according to the published reports (22–24). Referring to the involvement of regional lymph node by GA, the cN stage is regarded as a significant prognostic factor (17, 25). A multicenter Italian study reported that lymph node metastasis was a significant prognostic factor leading to an incredibly poor prognosis in GA patients with more than 6 involved nodes (26). And Gao et al. reported that more advanced pathological N-stage was an independent factor of metachronous ovarian metastasis from GA after radical gastrectomy (27). Based on the above literature, we can infer that with the higher cN stages, the tumor tends to show more invasive performances, resulting in less possibility of surgical resection.

As another independent risk factor of unresectability of GA, GTV measured on CT has been widely discussed in tumor TNM staging (28–30). In detail, it might be a comprehensive indicator reflecting the invasion length, depth and tumor diameter, and those indices could reflect the degree of tumor invasions to a certain extent. For example, Roedl et al. put forward that one of the independent risk factors of metastatic diseases in gastric cancer was the GTV measured on PET-CT (31), and another literature showed that the GTV could be a predictive factor of lymphovascular invasion (12). Both published reports indicated that the larger volume of the tumor, the more likely to behave aggressively. In the current study, we found that GTV could be closely associated with resectability of GA for the first time.

As shown in our research, age could be a potential risk factor of unresectability of GA according to the univariate analysis. Our finding is consistent with the report by Hsieh et al., which depicted that GA tended to exhibit more aggressive behaviors in young patients than in old patients (32). However, our study demonstrated that age was not an independent risk factor of unresectability based on our multivariate analysis.

Our study demonstrated that cT stage could be a potential risk factor of unresectability as shown by the univariate analysis. This finding can be explained by the published study, which depicted that the CT volumetry could be correlated well with T categories in gastric cancer (12). However, our multivariate analysis illustrated that the cT stage of GA could not be an independent risk factor of unresectability, which might be explained by the interaction between GTV and cT stage.

Because the cN stage and GTV of GA could be independent risk factors of unresectability as depicted in our study, we discussed the stratified analysis of the GTV according to the cN stage to provide a new quantitative modality for identifying the resectability for the first time. The N-stage based GTV performed well in determining the resectability of GA in stages cN_0-3_, cN_2_ and cN_3_. All the AUCs obtained by our ROC analyses of N-stage based GTV were greater than 0.85, suggesting a good performative assessment on resectability of GA. Our study subsequently validated the performance of the previous ROC models of N-stage based GTV for differentiating between resectable and unresectable GA in internal and external validation cohorts, and obtained good agreements, indicating that our ROC models could be reliable for determining the resectability. In general, the innovation of our article is that we have reported quantitative ROC models based on the combination of GTV and N-stage to preoperatively determine the resectability of GA for the first time. The clinical significance of the quantitative models could help preoperatively identify the resectability of GA, especially the resectability of gastric cancer without distant metastasis for treatment decision making.

There are several limitations in our study. Firstly, CT has been a tool with high sensitivity in detecting distant metastatic diseases including peritoneal disease, which makes it possible to determine resectability without measuring GTV. In addition, patients with N_1_ disease, all patients with T_3_ disease and most patients with T_2_ disease should be undergoing laparoscopy before attempted surgical resection as part of NCCN guidelines, indicating that the GTV prediction might be obviated. Despite the limitations, our GTV prediction could be an additional method to preoperatively identify the resectability of GA without distant metastasis. Secondly, in the cases with the tumor staged as cN_0-3_ and cN_2_, the specificity (between 0.6 to 0.7) is not satisfactory despite the sensitivity of more than 0.8. We will perform the relevant further study in the future to improve the sensitivity and specificity. Thirdly, microsatellite unstable gastric cancer has been reported to be stage-dependent, being the highest in node-negative disease (up to about 20%) and the lowest in metastatic disease (<5%) (33), suggesting that patients with resectable tumor may have large GTV. Our GTV prediction may not be suitable for determining resectability of microsatellite unstable gastric cancer but for identifying resectability of tumors without microsatellite unstability. We will perform the relevant study in the future. Fourth, the GTV of GA was obtained by manually sketching the abnormally thickened gastric wall in our study. Compared with machine learning algorithm, manual drawing may cost more time. However, the excellent results of inter- and intra- measurement of GTV in our study suggest that the manual drawing could be reliable. Fifth, our research is a retrospective study. We will focus on the thresholds of GTV in this study to determine the resectability of GA in our future prospective studies.

In summary, we found that the GTV and cN stage of GA could be associated with the resectability, and the N-stage based GTV can obtain a good performance on determining the resectability. The gross tumor volume cutoffs of 21.81 cm^3^, 21.70 cm^3^ and 36.93 cm^3^ may help estimate the resectability of GA in stage cN_0-3_, stage cN_2_ and stage cN_3_, respectively. We hope that our findings could be helpful for the quantitative determination of the resectability of GA for appropriate and precise treatment decision making in the future.

## Data availability statement

The raw data supporting the conclusions of this article will be made available by the authors, without undue reservation.

## Ethics statement

The studies involving human participants were reviewed and approved by the institutional ethics committee of Affiliated Hospital of North Sichuan Medical College. The patients/participants provided their written informed consent to participate in this study. Written informed consent was obtained from the individual(s) for the publication of any potentially identifiable images or data included in this article.

## Author contributions

T-wC, X-mZ, H-yZ, and RL proposed the study. Z-yY, DG, ZT, JO, K-yL, X-qC, DY, and L-lY performed the research, and collected the data. Z-yY, DG, and ZT analyzed the data and wrote the draft. All authors contributed to the interpretation of the study and to further drafts. All authors read and approved the final manuscript. T-wC is the guarantor.

## Conflict of interest

The authors declare that the research was conducted in the absence of any commercial or financial relationships that could be construed as a potential conflict of interest.

## Publisher’s note

All claims expressed in this article are solely those of the authors and do not necessarily represent those of their affiliated organizations, or those of the publisher, the editors and the reviewers. Any product that may be evaluated in this article, or claim that may be made by its manufacturer, is not guaranteed or endorsed by the publisher.

## Abbreviations

GA, gastric adenocarcinoma; CT, computed tomography; GTV, gross tumor volume; TC, training cohort; IVC, internal validation cohort; EVC, external validation cohort; ICC, Intraclass correlation coefficient; ROC, receiver operating characteristic; NCCN, National Comprehensive Cancer Network; AUC, area under the receiver operating characteristic curve; PPV, Positive predictive value; NPV, Negative predictive value.
